# Comparison of Pregnancy Rate and Live Birth Rate of Intracytoplasmic Sperm Injection Cycles Using Fresh Versus Frozen–Thawed Testicular Sperm

**DOI:** 10.1155/ogi/5544073

**Published:** 2025-11-10

**Authors:** Zeyad Mohammed Abualiat, Joud Sami Makki, Shahad Mubarak Aljebeli, Rawan Othman Bamousa, Orjowan Zuhair Alamri, Alwaten Fahad Alabdullah, Haya Al Fozan

**Affiliations:** ^1^Department of Obstetrics & Gynecology, King Abdulaziz Medical City, Ministry of National Guard Health Affairs, Riyadh, Saudi Arabia; ^2^Department of Obstetrics & Gynecology, King Abdullah International Medical Research Center (KAIMRC), Ministry of National Guard Health Affairs, Riyadh, Saudi Arabia; ^3^Department of Obstetrics & Gynecology, College of Medicine, King Saud bin Abdulaziz University for Health Sciences, Riyadh, Saudi Arabia

**Keywords:** fresh sperms, frozen sperms, intracytoplasmic sperm injection, pregnancy rate, Saudi Arabia

## Abstract

**Objectives:**

The primary objective of the study is to compare the rate of pregnancy and live birth rate in infertile couples with nonobstructive azoospermia (NOA) treated with either fresh or frozen sperms in intracytoplasmic sperm injection (ICSI) cycles. It is already known that the use of frozen sperm in ICSI cycles is considered favorable and is reported to have no significant difference in outcomes when compared to fresh sperm. However, there is still an ongoing debate about the superiority of fresh or frozen–thawed spermatozoa in men with NOA, particularly in the context of Saudi Arabia where this subject has not been previously investigated.

**Design:**

This retrospective cohort study was conducted at the in vitro fertilization (IVF) unit at KAMC-R, Ministry of National Guard Hospital Affairs (MNGHA) in Riyadh, Saudi Arabia. It includes couples who underwent ICSI cycles throughout four years (2019–2022), provided that they had no female infertility causes. A checklist was prepared to collect data from patients' medical records.

**Participants, Setting, and Methods:**

This retrospective cohort study analyzed data from 230 infertile couples who underwent ICSI cycles between 2019 and 2022. We compared pregnancy, live birth, and overall success rates in cycles using fresh versus frozen–thawed testicular sperm, controlling for factors like age, infertility type, and hormone levels. Statistical analysis included chi-square tests, Fisher's exact tests, Student's *t* tests, and Mann–Whitney tests to compare outcomes between groups.

**Results:**

A total of 231 women were included. Their mean ± standard deviation (SD) age was 31.5 ± 5.4. Almost two-thirds (67.5%) had primary infertility. Regarding the type of sperms used in intracytoplasmic sperm injection, fresh sperms represented 57.1%, while the remaining 42.9% were frozen sperms. Rates of pregnancy, live births, ectopic pregnancy, and abortion were 32.5%, 23.5%, 3.5%, and 5.7%, respectively. Overall, the success rate of ICSI was 29.8%. The pregnancy rate was significantly higher using fresh spermatozoa in ICSI than frozen spermatozoa (37.9% vs. 25.3%), *p*=0.043. Similarly, live births and overall successful outcome rates of using fresh spermatozoa in ICSI were significantly higher than those of frozen spermatozoa (28.8% and 34.8% vs. 16% and 22.6%), *p*=0.025 and 0.048, respectively.

**Limitations:**

The study demonstrated an association between fresh sperm use and higher success rates, but it did not prove causality.

**Conclusion:**

When both fresh and frozen sperm were used, the rates of pregnancy, live birth, and overall success in ICSI were higher in fresh sperm. Additionally, younger males and females showed a greater likelihood of successful ICSI outcomes. There is a potential benefit of using fresh sperm in ICSI for this specific patient group but highlights the need for further investigation to solidify these findings and to explore the reasons behind the observed differences. This study adds to the growing body of knowledge on ICSI for men with NOA and emphasizes the need for further research to refine clinical practice and improve outcomes for couples facing infertility.

## 1. Introduction

Infertility is characterized by the inability to achieve a clinical pregnancy in sexually active couples following 1 year or more of regular intercourse without contraceptive methods [1]. Azoospermia is one of the leading male-factor causes of infertility, impacting 10%–15% of the infertile males. It is characterized by the complete absence of sperm in the ejaculate after centrifugation of two specimens of semen [2, 3].

Azoospermia has two types: obstructive azoospermia (OA) and nonobstructive azoospermia (NOA). OA is produced secondary to failure of transportation of sperms from the testis to the urethra; on the other hand, NOA, which is more common, results from failure in spermatogenesis due to intrinsic testicular defect; however, in some men, focal areas of sperm production exist [4, 5].

Effective treatment for NOA is frequently done through two steps: microsurgical testicular sperm extraction (micro-TESE), which is followed by intracytoplasmic sperm injection (ICSI). Testicular biopsy can be done on the day of oocyte retrieval, and fresh sperm can be utilized to fertilize the oocytes [6]. The sperm recovery rate of this procedure ranged between 16.7% and 63% in cases with NOA [7].

In case of failure of pregnancy after micro-TESE followed by ICSI, another testicular biopsy will be needed for the next cycle. However, repeated TESE could not achieve complete recovery [8]. Therefore, cryopreservation of sperm is essential to avoid repeated biopsies from the testis for each ICSI cycle. Using frozen–thawed testicular tissue and testicular spermatozoa was associated with successful clinical pregnancy and is generally recommended in such patients [9].

The literature review indicated that most studies found no notable difference in outcomes (fertilization rate (FR), implantation rate (IR), and clinical pregnancy rate (CPR)) between the use of fresh and frozen–thawed testicular sperm [10]. However, a study by Park et al. included a large sample size that reported statistical differences in the IR and CPR between fresh and frozen–thawed testicular sperm [11]. Another study that compared the use of fresh and frozen–thaw spermatozoa in azoospermic patients with nonmosaic Klinefelter syndrome (KS) revealed higher fertilization and CPRs with fresh spermatozoa, with no differences regarding implantation or newborn rates [12].

Although it has been reported in the literature that using frozen sperm in ICSI cycles is a favorable method with no significant difference with regard to the outcome with fresh sperm, arguments about the use of fresh or frozen–thawed spermatozoa in men with NOA still exist and need further studies, particularly in Saudi Arabia as this subject was not previously investigated. The objective of this study is to compare the rate of pregnancy and live birth rate in infertile couples with NOA treated with either fresh or frozen sperms in ICSI cycles throughout two years (2019–2022) at KAMC-R infertility unit, Ministry of National Guard Health Affairs (MNGHA), Riyadh.

## 2. Materials and Methods

### 2.1. Study Design

This retrospective cohort study was conducted at the In Vitro Fertilization (IVF) unit at KAMC-R, MNGHA in Riyadh.

### 2.2. Study Population

Couples who underwent ICSI cycles throughout four years (2019–2022) at the IVF unit, Ministry of National Guard Hospital, Riyadh, with male factor as a cause of the infertility were eligible for inclusion in the study. However, female infertility causes, normal semen analysis, were excluded from the study.

### 2.3. Data Collection Instrument and Procedure

A checklist was prepared to collect data from patients' medical records. It included information about the wife's age, parity, type of infertility (primary and secondary), gynecological history, husband's age, husband's smoking status, husband's sexual dysfunction, type of used sperms in ICSI (fresh or frozen), and pregnancy after ICSI and live birth.

### 2.4. Statistical Analysis

Statistical analysis was conducted using SPSS software, version 28. Categorical data were presented as frequencies and percentages. In contrast, continuous variables were represented in mean, median range, interquartile range (IQR), and standard deviation (SD), depending on the variable distribution. Chi-square test/Fisher's exact tests were applied for the association between categorical variables. The Students' *t* tests and Mann–Whitney tests were employed to compare a numerical variable between two distinct groups, with a significance level set at a *p* value below 0.05.

### 2.5. Statement of Ethics

Study approval statement: This study was reviewed and approved by the Local Research and Ethical Committee, KAIMRC, at the MNGH, Riyadh, Saudi Arabia, under IRB number (IRB/1329/23). Because of the retrospective nature of the study, written informed consent was not required, and the study has been granted an exemption from requiring written informed consent from the same committee.

## 3. Results

A total of 230 women who underwent ICSI were included in the study.  Table 1 summarizes their characteristics. Their age ranged between 19 and 44 years with an arithmetic mean ± SD of 31.5 ± 5.4. Almost two-thirds were nulliparous 69.5% with primary infertility of 67.5%. Most were overweight (BMI 25–29.9) of 34.2% or obese (BMI of 30–34.9) of 35.9%. The median (IQR) of antral follicle count was 9 (5–12). Follicular stimulating hormone levels ranged between 0.99 and 12.3 mIU/mL with an arithmetic mean ± SD of 10.9 ± 18.7 mIU/mL.

Characteristics of husbands are summarized in Table 2. Their age ranged between 22 and 72 years with an arithmetic mean ± SD of 39.0 ± 9.9 years. Almost two-thirds (67.8%) had primary infertility. Percentage of healthy sperm morphology was 26.5%. Prevalence of smoking was 29.6%, whereas that of sexual dysfunction and obstructive azoospermia was 12.6% and 22.6%, respectively. Regarding the type of sperms used in ICSI, fresh sperms represented 57.4% of the total, while the remaining 42.6% were frozen sperms.

Table 3 shows that the pregnancy rate was significantly higher using fresh spermatozoa in ICSI than frozen spermatozoa (37.9% vs. 25.3%); *p* = 0.043. Similarly, live births and overall successful ICSI trials, defined as cycles that resulted in Clinical pregnancy include intrauterine gestation, ectopic pregnancy, or miscarriage, rates of using fresh spermatozoa in ICSI were significantly higher than those of frozen spermatozoa (28.8% and 34.8% vs. 16% and 22.6%), *p* = 0.025 and 0.048, respectively. Also, the rate of abortion was higher, although not significant, among women who used fresh than those who used frozen sperm.

### 3.1. Factors Associated With Successful Intracytoplasmic Sperm Injection

Women-related factors: The age of women with successful ICSI was significantly lower than those with failed ICSI (29.3 ± 5.1 vs. 32.5 ± 5.3), *p* < 0.001. Women with higher antral follicle count were likely to have successful ICSI, *p*=0.049 for the right side (Table 4).

Husband-related factor: The age of husbands whose women had successful ICSI was significantly lower than those with failed ICSI (36.8 ± 8.5 vs. 40.1 ± 10.4), *p* < 0.001.  Table 5 shows a regression analysis of factors associated with successful ICSI trials.

## 4. Discussion

A review of the literature uncovered a limited number of relatively recent studies that explored the disparities in ICSI outcomes between fresh and frozen sperm usage. Notably, to our knowledge, none of these studies were conducted within Saudi Arabia, suggesting a unique opportunity for localized research in this area.

Similar to our finding, a study by Gunby et al. found a significantly higher pregnancy rate, live birth rate, and overall successful rate of ICSI using fresh sperms compared to frozen sperms [13]. However, several previous studies did not report a significant difference between fresh or frozen sperms associated with higher pregnancy rates (14–19). Contrary to our findings, Zargar et al. [14], Wang et al. [15], and Roque et al. [16] observed that successful fertility and pregnancy rates were higher in IVF by frozen sperms compared to fresh sperms. In the current study, the rate of abortion was higher, although not significant, among women who used fresh (6.1%) than those who used frozen sperm (5.2%). Contrary to that, in the United States, the pregnancy loss rate was significantly higher in the frozen group than in the fresh group (33% versus 5.9%) [17].

In a recent study by Miller et al., which compared the outcomes of ICSI using fresh versus frozen–thawed testicular sperm in men with NOA, no significant disparities were observed in fertilization outcome, cleavage rate, reasonable embryo rate, neither the rate of the implantation, clinical pregnancy, or live birth rates as observed in the two groups. They reported that although the live birth rate was not statistically different when frozen versus fresh sperm was utilized, there was a clinically significant trend towards improved outcomes with fresh sperm [18]. However, Jin et al. revealed that the FR of the experimental group, which used frozen–thawed sperm, was significantly higher than that of the control group, which used fresh PESA sperm (84.1% vs. 73.3%), while no significant differences were reported with regard to IR, CPR, and newborn weights. In the experimental group, eight clinical pregnancies were achieved, including five live deliveries and three ongoing pregnancies; 37 clinical pregnancies, including 30 deliveries with only one fetal death; three ongoing pregnancies; and four abortions in the control group [19]. Equally important is the support that different research trends are possessed in light of facts already established [20–22]. It stresses the need for further accumulating evidence in this fast-developing area to improve treatment and clinical practices. In this regard, the opposing results in these reports point to the necessity for expanding the studies to investigate the factors and conditions under which either fresh or frozen sperm can be most effective for each patient group.

In the present study, successful ICSI was associated with younger male and female ages and higher antral follicle count. It has been previously concluded that antral follicle count is better than anti-Mullerian hormone level in predicting poor ovarian response, and the only significant determinant for successful ICSI was age [19, 23, 24]. This is the only Saudi study that compared the pregnancy and live birth rates between utilizing fresh and frozen sperms in ICSI.

### 4.1. Limitations

However, some limitations should be mentioned, including the study's retrospective nature, and being a single-center experience. The study population was drawn from a single IVF unit in Saudi Arabia, potentially limiting its generalizability to other populations. The study relied on existing data, which was not collected with the specific aim of addressing the research question. The study can only show associations between the use of fresh sperm and better outcomes, not cause-and-effect. While the study included 230 couples, this is relatively small. These limitations highlight the need for further research.

## 5. Conclusions

The pregnancy rate, live birth rate, and overall successful rate of ICSI were higher when fresh compared to frozen sperm were utilized. Further, a more extensive prospective multicentric study is recommended to clarify this issue better in Saudi Arabia. In addition, it is suggested that both males and females start ICSI at earlier ages to increase the chance of successful outcomes.

## Figures and Tables

**Table 1:** Characteristics of women who underwent intracytoplasmic sperm injection (*N* = 230).

Variables	Frequency	Percentage
Age in year		
Range	19–44	
Mean ± SD	31.5 ± 5.4	
Parity		
Nulliparous	160	69.6
One	43	18.7
Two/more	27	11.7
Type of infertility		
Primary	156	67.8
Secondary	74	32.2
Body mass index		
Underweight	5	2.2
Normal	63	27.4
Overweight	79	34.3
Obesity	83	36.1
Antral follicle count (median “IQR”)	Median	IQR
Right	9	5–12
Left	9	5–12
Follicular stimulating hormone (mIU/mL) (*n* = 189)		
Range	0.99–123	
Mean ± SD	10.9 ± 18.7	

Abbreviation: SD, standard deviation.

**Table 2:** Characteristics of the husbands who underwent intracytoplasmic sperm injection (*N* = 230).

Variables	Frequency	Percentage
Age in years		
Range	22–72	
Mean ± SD	39.0 ± 9.9	
Type of infertility		
Primary	156	67.8
Secondary	74	32.2
Healthy sperm morphology % (*n* = 61)		
0	12	5.2
1	8	3.5
2	29	12.6
> 2	12	5.2
Nonhealthy	169	73.5
Karyotyping		
Normal	226	98.3
Abnormal	4	1.7
Microdeletion		
No	229	99.6
Yes	1	0.4
Smoking		
No	162	70.4
Yes	68	29.6
Sexual dysfunction		
No	201	87.4
Yes	29	12.6
Type of azoospermia		
Nonobstructive	178	77.4
Obstructive	52	22.6

**Table 3:** Comparison of pregnancy, live birth, and successful rates of intracytoplasmic sperm injection between fresh and frozen sperms (*n* = 230).

	Type of used sperms		*p* value
	Fresh *N* = 132 (57.4) *N* (%)	Frozen *N* = 98 (42.6) *N* (%)	
Pregnancy			
No	82 (62.1)	73 (74.7)	0.043
Yes	50 (37.9)	25 (25.3)	
Live births			
No	94 (71.2)	83 (84.0)	0.025
Yes	38 (28.8)	15 (16.0)	
Abortion			
No	124 (93.9)	93 (94.8)	0.770
Yes	8 (6.1)	5 (5.2)	
Outcome			
Failed	86 (65.2)	77 (77.4)	0.048
Successful	46 (34.8)	21 (22.6)	

**Table 4:** Women-related factors associated with successful intracytoplasmic sperm injection (*n* = 230).

	Intracytoplasmic sperm injection		*p* value
	Failed *N* = 158/68.7 *N* (%)	Succeeded *N* = 72/31.3 *N* (%)	
Age in year			
Mean ± SD	32.5 ± 5.3	29.3 ± 5.1	< 0.001^∗^
Parity			
Nulliparous	109 (47.4)	51 (22.2)	0.904^∗∗^
One	29 (12.6)	14 (6.1)	
Two/more	20 (8.7)	7 (3.0)	
Type of infertility			
Primary (*n* = 156)	106 (70.2)	50 (21.7)	0.991^∗∗^
Secondary (*n* = 74)	52 (70.3)	22 (9.7)	
Body mass index			
Underweight (*n* = 6)	4 (1.7)	7 (3.0)	0.675^∗∗^
Normal (*n* = 62)	40 (17.4)	22 (9.6)	
Overweight (*n* = 77)	55 (23.9)	22 (9.6)	
Obesity (*n* = 80)	59 (25.7)	21 (9.1)	
Antral follicle count			
Right (median “IQR”)	9 (5–12)	10 (5–15)	0.049^ⱶ^
Left (median “IQR”)	8 (5–11)	10 (6–12)	0.120^ⱶ^
Follicular stimulating hormone			
Mean ± SD	12.05 ± 20.93	8.22 ± 12.34	0.203

Abbreviation: NA, not applicable.

^∗^Independent two-sample *t*-test.

^∗∗^Chi-square test.

^ⱶ^Mann–Whitney test.

**Table 5:** Husband-related factors associated with successful intracytoplasmic sperm injection.

	Intracytoplasmic sperm injection		*p* value
	Failed *N* = 158 *N* (%)	Succeeded *N* = 72 *N* (%)	
Age in year			
Mean ± SD	40.1 ± 10.4	36.8 ± 8.5	0.025
Type of infertility			
Primary	106 (46.1)	50 (21.7)	0.200
Secondary	52 (22.6)	22 (9.6)	
Karyotyping			
No	154 (67)	72 (31.3)	0.320
Yes	4 (1.7)	0 (0.0)	
Microdeletion			
No	157 (68.3)	72 (31.3)	0.701
Yes	1 (0.4)	0 (0.0)	
Smoking			
No	114 (49.6)	49 (21.3)	0.346
Yes	44 (19.1)	23 (10)	
Sexual dysfunction			
No	135 (58.7)	66 (28.7)	0.467
Yes	23 (10)	6 (2.6)	

## Data Availability

The data will be available on request from the corresponding authors.

## Nomenclature

OA: Obstructive azoospermia
NOA: Nonobstructive azoospermia
Micro-TESE: Microsurgical testicular sperm extraction
ICSI: Intracytoplasmic sperm injection
FR: Fertilization rate
IR: Implantation rate
CPR: Clinical pregnancy rate
IVF: In vitro fertilization
FTEPS: Frozen–thawed epididymal spermatozoa
FTTS: Frozen–thawed testicular spermatozoa
PESA: Percutaneous epididymal sperm aspiration
SD: Standard deviation
SPSS: Statistical package for social sciences
